# Alpha-B-Crystallin Effect on Mature Amyloid Fibrils: Different Degradation Mechanisms and Changes in Cytotoxicity

**DOI:** 10.3390/ijms21207659

**Published:** 2020-10-16

**Authors:** Olga V. Stepanenko, M. I. Sulatsky, E. V. Mikhailova, Olesya V. Stepanenko, O. I. Povarova, I. M. Kuznetsova, K. K. Turoverov, A. I. Sulatskaya

**Affiliations:** 1 Laboratory of Structural Dynamics, Stability and Folding of Proteins, Institute of Cytology, Russian Academy of Sciences, 4 Tikhoretsky ave., 194064 St. Petersburg, Russia; sov@incras.ru (O.V.S.); 4evamkh@gmail.com (E.V.M.); lvs@incras.ru (O.V.S.); olp@incras.ru (O.I.P.); imk@incras.ru (I.M.K.); ansul@mail.ru (A.I.S.); 2 Laboratory of Cell Morphology, Institute of Cytology, Russian Academy of Sciences, 4 Tikhoretsky ave., 194064 St. Petersburg, Russia; m_sulatsky@mail.ru; 3 Peter the Great St.-Petersburg Polytechnic University, Polytechnicheskaya 29, 195251 St. Petersburg, Russia

**Keywords:** chaperones-based therapy of amyloidosis, heat shock protein alpha-B-crystallin, mechanisms of amyloid fibrils degradation, fragmentation and decompactization of amyloid fibers, stability and cytotoxicity of amyloid fibrils

## Abstract

Given the ability of molecular chaperones and chaperone-like proteins to inhibit the formation of pathological amyloid fibrils, the chaperone-based therapy of amyloidosis has recently been proposed. However, since these diseases are often diagnosed at the stages when a large amount of amyloids is already accumulated in the patient’s body, in this work we pay attention to the undeservedly poorly studied problem of chaperone and chaperone-like proteins’ effect on mature amyloid fibrils. We showed that a heat shock protein alpha-B-crystallin, which is capable of inhibiting fibrillogenesis and is found in large quantities as a part of amyloid plaques, can induce degradation of mature amyloids by two different mechanisms. Under physiological conditions, alpha-B-crystallin induces *fluffing* and *unweaving* of amyloid fibrils, which leads to a partial decrease in their structural ordering without lowering their stability and can increase their cytotoxicity. We found a higher correlation between the rate and effectiveness of amyloids degradation with the size of fibrils clusters rather than with amino acid sequence of amyloidogenic protein. Some external effects (such as an increase in medium acidity) can lead to a change in the mechanism of fibrils degradation induced by alpha-B-crystallin: amyloid fibers are fragmented without changing their secondary structure and properties. According to recent data, fibrils *cutting* can lead to the generation of *seeds* for new *bona fide* amyloid fibrils and accelerate the accumulation of amyloids, as well as enhance the ability of fibrils to disrupt membranes and to reduce cell viability. Our results emphasize the need to test the chaperone effect not only on fibrillogenesis, but also on the mature amyloid fibrils, including stress conditions, in order to avoid undesirable disease progression during chaperone-based therapy.

## 1. Introduction

Ordered aggregates of proteins, amyloid fibrils, are a marker of many serious diseases, such as Alzheimer’s, Parkinson’s, Huntington’s disease, a number of prion diseases, and diabetes [1,2,3,4,5,6,7]. Recent studies have shown that the formation of amyloid fibrils can be not only a pathological process. Amyloid-like fibrils are involved in vital processes [8,9,10,11,12]. This means that there are special mechanisms for the assembly/disassembly of amyloid fibrils, which make it possible to ensure their functional activity and elimination, preventing the development of pathological conditions. Chaperones may contribute to these processes as they mainly function to maintain the correct native tertiary or quaternary structure of proteins, as well as to form and dissociate protein complexes [13,14,15].

A lot of works investigated the effect of chaperones on fibrillogenesis. Initially, it was believed that the action of chaperones was normally aimed solely at preventing the formation of amyloid fibrils. The effect is exemplified by small heat shock proteins (sHSP), which function as molecular chaperones, ATP-independently preventing stress-induced aggregation of partially denatured or misfolded proteins and facilitating their return to native conformation [16]. Thus, sHSP normally protect the structure of the protein and maintain its functional activity, thereby preventing disease development. Furthermore, other ways of inhibiting amyloid fibrils’ formation were shown. It was found that the chaperone-like protein 14-3-3η was unable to bind to the monomer of the amyloidogenic protein alpha-synuclein, but could interact with amyloid oligomers formed from this protein and prevent its further aggregation [17]. Hsp70 chaperone also suppressed the formation of alpha-synuclein amyloid fibrils at the stage of amyloid oligomer formation [18]. Alpha-B-crystallin was able to inhibit the proliferation of fibrils formed from Abeta-peptides (1–40) and (1–42) by binding to the amyloid seeds (short fibrillar fragments) [19,20].

Despite the fact that a large number of works indicate the inhibitory effect of chaperones on the formation of amyloid fibrils, there are some articles that show the ability of chaperones to accelerate this process or some of its stages. For example, it was shown that the GroEL chaperonin accelerated the formation of protofibrils of the Het-s prion protein, and then interacted with them, thereby preventing the formation of full-length mature fibrils [21]. In a number of other works, the possibility of activation of the process of amyloid fibrils formation and a reduction in the duration of the lag phase by chaperones at their substoichiometric concentrations, in comparison with fibrillogenesis in the absence of chaperones, has been shown [22,23]. Nevertheless, as we already noted, according to the results of most studies of the chaperones and chaperon-like proteins’ effect on fibrillogenesis, these proteins can be specifically and effectively targeted to slow or prevent amyloid disease progression (see, for example, the review [24]).

At the same time, the effect of chaperones and chaperon-like proteins on mature amyloid fibrils is currently a much less studied problem. However, given the fact that amyloidosis is often detected at late stages of the diseases, when a large number of amyloid plaques accumulate in the patient’s body, solving this problem is of high importance for the treatment of progressive amyloidosis. A number of works show either no effect [25,26,27,28] or weak effect, requiring 30-day incubations with chaperones for 50% disassembly of amyloids [29]. Currently available literature data proving the interaction of chaperones with mature amyloid fibrils [19,30,31,32,33] do not provide grounds for an unambiguous conclusion about the mechanisms of chaperone action on these protein aggregates, about the universality of these mechanisms, and also about what determines the effectiveness of the chaperone action. Efficient disassembly of amyloid aggregates in vitro on a physiologically significant time scale (from minutes to hours) has now been demonstrated in a very small number of studies [31]. Based on data on the different effects of chaperones on fibrillogenesis, it can be assumed that their influence on mature amyloid fibrils can also be different. In this regard, the aim of our work was to compare the influence of the chaperon-like protein on mature amyloid fibrils formed from various amyloidogenic proteins and their cytotoxicity, to find out what affects the mechanism and efficiency of chaperon action, as well as what occurs when the external conditions change. As a protein with chaperone activity, we chose the small heat shock protein alpha-B-crystallin known to be involved in inhibiting apoptosis and contributing to intracellular architecture as well as in the binding misfolded proteins and preventing protein aggregation [34,35]. Alpha-B-crystallin is found in large quantities in the body of patients as a part of amyloid plaques [19] and is able to inhibit the proliferation of amyloid fibrils formed from various amyloidogenic proteins [19,20,36]. In particular, it has been previously shown that alpha-B-crystallin not only increases the lag phase of alpha-synuclein aggregation, slowing down the formation of prefibrillar intermediates, but also inhibits the growth phase of amyloid fibers [20,37].

## 2. Results

### 2.1. The Studied Amyloid Fibrils Formed from Lysozyme and Beta-2-Microglobulin Have A Similar Structure and Morphology, But Differ in the Size of Their Clusters

As an object of the study, we decided to choose the amyloid fibrils which formation and accumulation in various tissues and organs was not only a marker of pathology, but also a direct cause of deterioration in the quality of patients’ life. Such diseases are light chains amyloidosis (AL-amyloidosis) [38,39], hereditary lysozyme systemic amyloidosis (ALys amyloidosis) [40], dialysis-related amyloidosis (DRA) [41], etc. The reasons for the accumulation of amyloid fibrils in such diseases are different. In particular, it can be a change in the secondary or tertiary structure of proteins, including as a result of mutations in their amino acid sequence (for example, ALys amyloidosis) or their synthesis in abnormal malignant cells (for example, AL-amyloidosis); accumulation of high concentrations of protein in the body, including as a result of medical procedures (for example, DRA, Insulin-derived amyloidosis), etc., which increases proteins’ tendency to aggregate with the formation of amyloid fibrils. Lysozyme (ALys amyloidosis) and beta-2-microglobulin (DRA), as proteins with significantly different secondary and tertiary structures and stability, were chosen by us as target proteins. Amyloid fibrils formed from these proteins were prepared by previously developed approaches [42,43].

**Figure 1:**
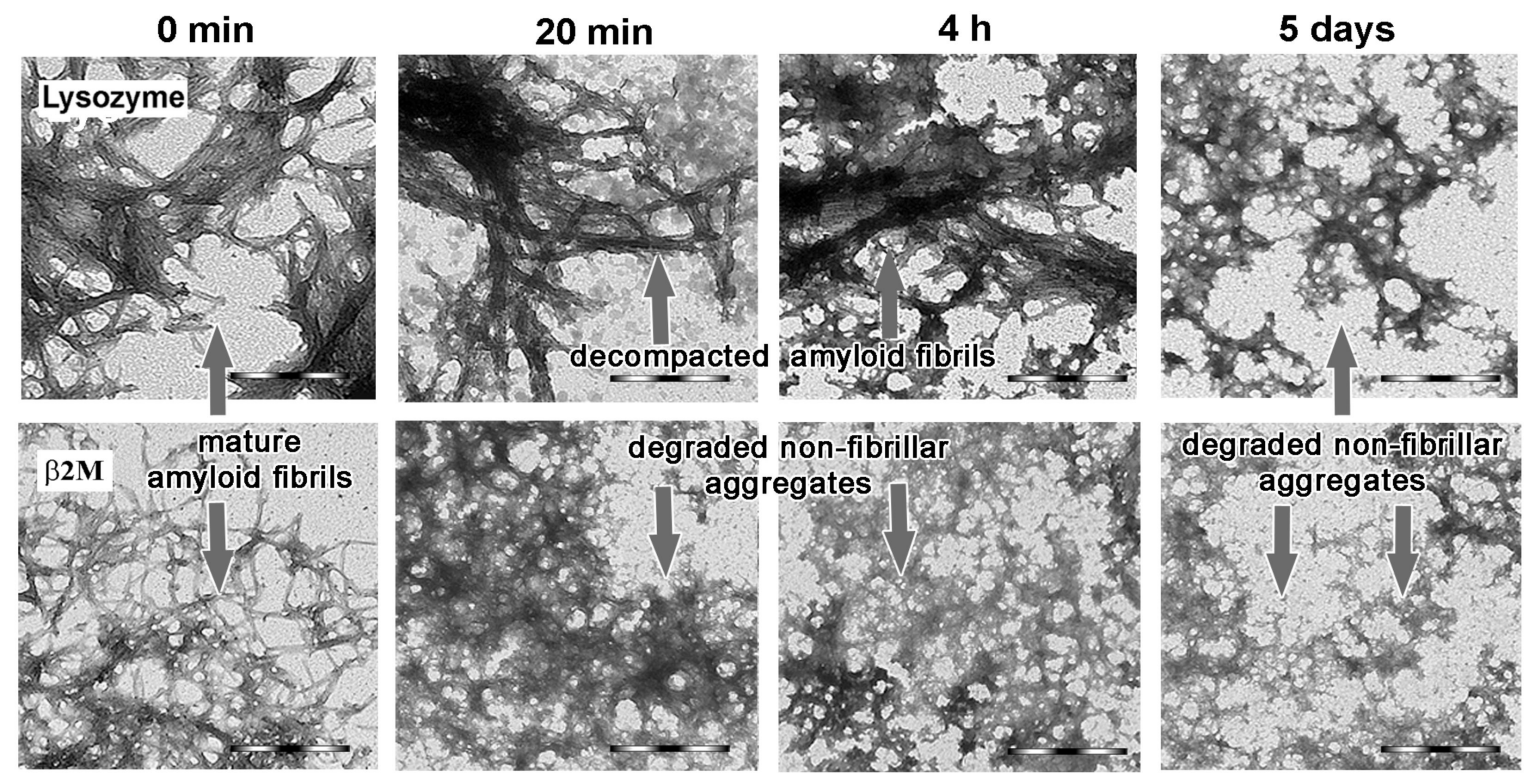
Alpha-B-crystallin-induced *fluffing* of amyloid fibrils under neutral conditions. TEM images of degradation of the mature amyloid fibrils prepared from lysozyme at pH 7 (**top row**) and from beta-2-microglobulin (β2M) at pH 2 (**bottom row**) in the presence of alpha-B-crystallin. The time after the addition of chaperone to fibrils is indicated above the panels. The experiments were conducted under physiological conditions (pH 7.4) and the molar ratio of fibrils to alpha-B-crystallin was kept at 1:0.5. Scale bars are equal to 1 µm.

According to the literature, pH optimum of the functional activity of alpha-B-crystallin is in the range from 7 to 8 [30,44]. In this regard, prepared in the special conditions amyloid fibrils were transferred to a buffer with pH 7.4. The stability of amyloid fibrils under new conditions in the absence of external influences was monitored during all experiments: the morphology of amyloid fibrils, their size and secondary structure were preserved at least during the course of the experiment with alpha-B-crystallin (Figure S1). TEM data indicated that mature amyloids formed from lysozyme and beta-2-microglobulin had a classic fibrillar structure, as well as a high tendency to interact with each other to form clots (Figure 1). It was also shown that, despite the difference in the structure of monomeric proteins, their fibrils had a similar secondary structure with a high content of beta-sheets forming the fibrillar core (Table 1). At the same time, a greater thickness of lysozyme fibrils and their denser “packing” into clusters compared to beta-2-microglobulin fibrils was observed (Figure 1).

In addition, the Rayleigh light scattering (RLS) value of fibrils indicated a larger size of lysozyme aggregates in comparison to beta-2-microglobulin fibrils (Table 2). The values of fluorescence anisotropy and parameter *A* (which is the ratio of fluorescence intensities at wavelengths of 320 and 365 nm and characterizes the change in the shape and position of the fluorescence spectrum) [46] of the investigated fibrils were relatively high. This is inherent of amyloid-forming proteins, tryptophan residues of which typically have a dense microenvironment.

### 2.2. Alpha-B-Crystallin Binds to the Entire Surface of the Amyloid Fiber

Alpha-crystallin is the main structural protein in the eye lens and its concentration is extremely high (about 450 mg/mL). Alpha-B-crystallin is mainly expressed in the eye lens, while alpa-B-crystallin is also expressed in other tissues, including the nervous system [47]. This protein accumulates in neurons and glia of the central nervous system under pathological conditions [48], becoming colocalized with aSyn in Lewy bodies. It was shown that alpha-B-crystallin is associated with myopathies, and neurodegenerative disorders including Parkinson’s, Alzheimer’s, and Creutzfeldt–Jakob diseases [49,50]. This protein is found in large quantities in the body of patients as a part of amyloid plaques [19]. It is assumed that the prominent increase in alpha-B-crystallin in pathological glia may be a response to stress [49] and its concentration in this case can be very different from the physiological one. In this regard, the accurate estimation of the concentration of alpha-B-crystallin and its ratio with the concentration of amyloid fibrils in an organism is very difficult (due to the variety of diseases, their pathogenesis, stress reactions of individual organisms, etc.).

In our work, we aimed to study a specific mechanism of alpha-B-crystallin’s effect on mature fibrils and selected the ratio and concentration of ligand and receptor so that we could detect noticeable changes in the amyloid fibrils structure for several days. We used mature amyloid fibrils at 0.1 mg/mL concentration (this concentration was optimal for determination of a wide range of physicochemical characteristics of fibrils and their visualization). We chose the molar ratio of fibrils to alpha-B-crystallin equal to 1/0.5 on the basis of literature analysis [19,20,51] and, using the results of our preliminary studies, which showed that at lower ratios (for example, when the molar ratio of fibrils to alpha-B-crystallin was 1/0.1), there was no pronounced effect of the chaperone for several days (Figure S2). However, this does not mean that alpha-B-crystallin has no effect on fibrils at lower chaperone concentrations and at a different chaperone/fibril ratio compared to those used in our work. We assume that using the other experimental conditions can change the effectiveness and rate of alpha-B-crystallin exposure, but does not change the mechanism of the chaperone influence.

According to the literature, alpha-B-crystallin in physiological conditions has a highly heterogeneous nature. The modeling based on the electron microscopy analysis of alpha-crystallin shows that the main population of the alpha-B-crystallin polydisperse complex is represented by oligomeric particles of slightly ellipsoidal shape with a diameter of about 13.5 nm and a molecular mass of about 700 kDa [52]. The results obtained by us are in a good agreement with the literature (Figure S3). As already noted, the physiological concentration of alpha-crystallin in the eye lens is extremely high, which can contribute to its pathological aggregation (cataract disease). However, we used the alpha-B-crystallin at a concentration of 0.07 mg/mL, which should not lead to any changes in its oligomeric status. The experimental results confirmed this assumption: the size of oligomers, their morphology and secondary structure in the control sample (without amyloid fibrils) did not change, at least at the time during which the experiment with amyloid fibrils was carried out (Figure S3).

The TEM data obtained immediately after the addition of alpha-B-crystallin to fibrils confirmed the binding of alpha-B-crystallin to fibrils. Figure S4 shows the interaction of alpha-B-crystallin aggregates with the surface of amyloid fibrils along their long axis that indicates high binding stoichiometry of chaperon to amyloid protein. According to the literature, the stoichiometry of the interaction of alpha-B-crystallin with amyloid fibrils formed from alpha-synuclein (which has a molecular weight close to the proteins studied in our work) ranges from 0.23 to 0.9 molecules of alpha-B-crystallin (results obtained using various approaches) per 1 molecule of amyloidogenic protein [33]. It was shown that alpha-B-crystallin binds to Abeta (1–40) and (1–42) fibrils with micromolar affinity, with a maximum molar binding ratio of 0.57 and 0.34 chaperon monomers per Abeta (1–40) and (1–42) monomer, respectively. Thus, the existing data on the stoichiometry of alpha-B-crystallin binding to fibrils formed from various proteins and having significantly different morphology are quite close (taking into account the high error in these values’ determination), which means that these parameters can also be valid in the case of alpha-B-crystallin with amyloid fibrils studied in the present work. It should be noted that high binding stoichiometry requires alpha-B-crystallin binding to the entire surface of the fibril, and not just to the ends of the fibrils (as suggested in some works), which is in good agreement with the binding model that we demonstrated using EM (Figure S4).

### 2.3. Alpha-B-Crystallin Induces Degradation of Mature Amyloid Fibrils with the Formation of Large Less Ordered Aggregates

**Figure 2:**
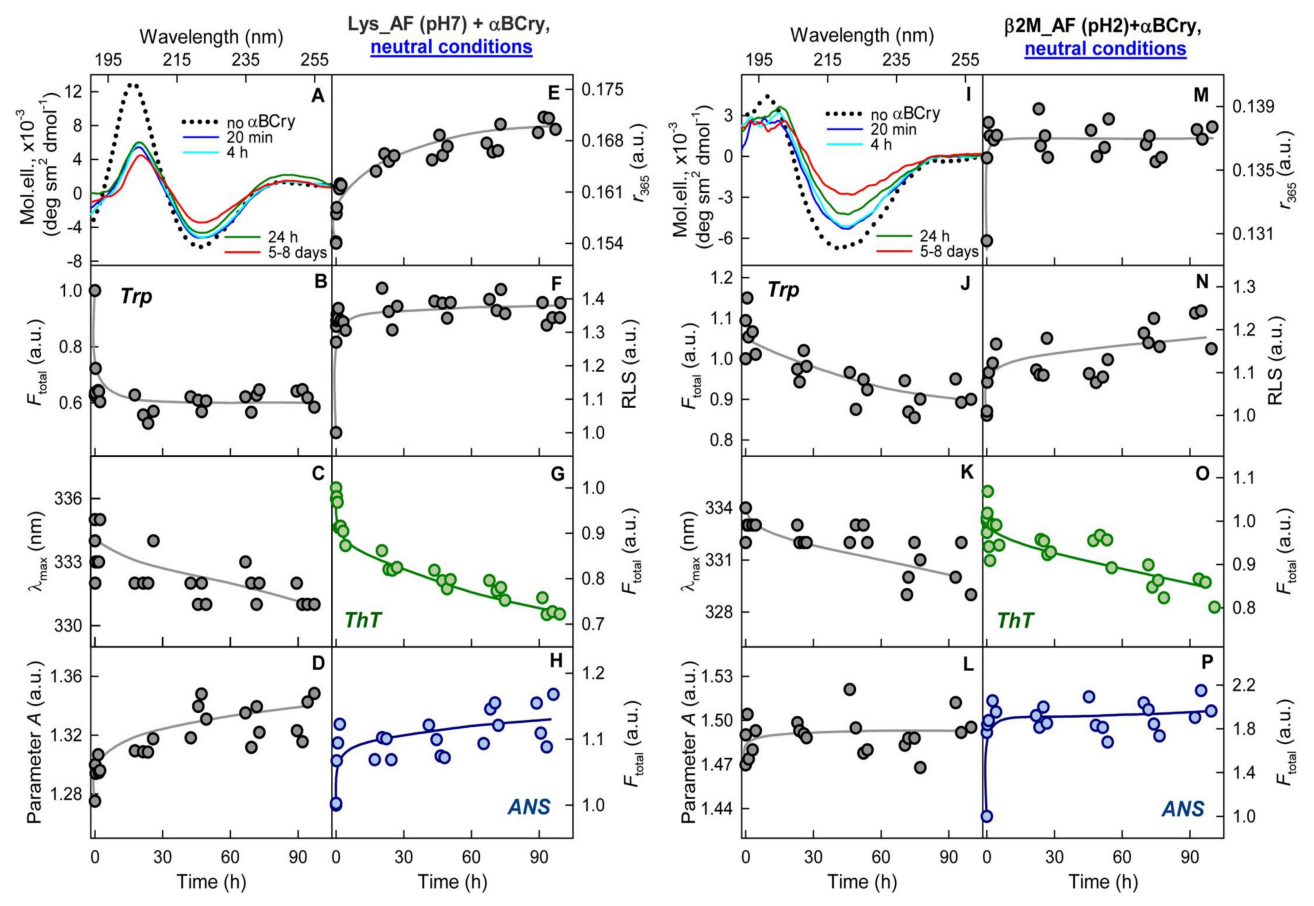
Structural changes of amyloid fibrils formed from lysozyme and beta-2-microglobulin in the presence of alpha-B-crystallin under neutral conditions. Amyloid fibrils were prepared from lysozyme at pH 7 (Lys_AF (pH 7), left panels) and from beta-2-microglobulin at pH 2 (β2M_AF (pH 2), right panels). Figure shows alpha-B-crystallin induced changes in (**A** and **I**) far UV CD spectra of amyloid fibrils; (**B** and **J**) integrated fluorescence intensity of amyloid fibrils at excitation wavelength of 295 nm; (**C** and **K**) wavelength of the fluorescence spectrum maximum of amyloid fibrils (λ_max_); (**D** and **L**) parameter *A* of amyloid fibrils, λ_ex_ = 295 nm; (**E** and **M**) fluorescence anisotropy (*r*) of amyloid fibrils, λ_ex_ = 295 nm, λ_em_ = 365 nm; (**F** and **N**) Rayleigh Light Scattering (RLS) of amyloid fibrils; (**G** and **O**) integrated fluorescence intensity of ThT in the presence of amyloid fibrils; (**H** and **P**) integrated fluorescence intensity of ANS in the presence of amyloid fibrils. The values at the panels (**B** and **J**, **F** and **N**, **G** and **O**, **H** and **P**) are normalized to the values for fibrils in the absence of alpha-B-crystallin. The experiments were conducted under physiological conditions (pH 7.4) and the molar ratio of fibrils to alpha-B-crystallin was kept at 1:0.5. The standard error of the mean is determined for a confidence interval of 0.95 and does not exceed 10%.

It was shown that the presence of alpha-B-crystallin in the sample induced degradation of the amyloid fibrils by *fluffing*, *unwinding* and *decompaction* of their structure (Figure 1). Decompaction, in turn, led to an increase in the size of aggregates, which was confirmed by an increase in the RLS of the samples (Figure 2F,N). In parallel with the decompaction of amyloid fibrils, the formed aggregates were partially degraded into smaller aggregates (Figure 1). Structural changes in amyloid fibrils were confirmed by changes in the intrinsic fluorescent characteristics of the samples (Figure 2). We observed a decrease in intrinsic fluorescence intensity, a shift in fluorescence spectra to short wavelengths, and an increase in fluorescence anisotropy and parameter *A* for amyloid fibrils formed from the both proteins (Figure 2B–E,J–M). Far-UV circular dichroism (CD) spectroscopy results indicated a decrease in the ordering of the secondary structure in the sample (Figure 2A,I, Table 1). In particular, far-UV CD spectra of lysozyme and beta-2-microglobulin fibrils at the beginning of the experiment had an apparent minimum around 220 nm, reflecting the essential content of beta-sheets in their structure [53,54]. This fact was confirmed using the CDPro software containing three popular methods (CONTIN, SELCON3, and CDSSTR) [55] and several basic sets of proteins with a known secondary structure (that include from 37 to 56 soluble, membrane, and denatured proteins with different contents of their secondary structure) (Table 1). It was shown that alpha-B-crystallin induced a significant decrease in minimum around 220 nm in the far-UV CD spectrum of both studied objects (Figure 2A) and, accordingly, a decrease in the ordered structure (Table 1).

The degradation process of amyloid fibrils was also confirmed by a decrease in the fluorescence intensity of the amyloid-specific dye thioflavin T (ThT) (Figure 2G,O), which is widely used to diagnose the occurrence and study the structure of amyloid fibrils [56,57,58,59]. Conversely, the fluorescence intensity of the hydrophobic probe 1-anilinonaphthalene-8-sulfonic acid, ANS, which integrates into hydrophobic cavities between protein associates forming large aggregates [60,61,62], as well as interacting with amyloid fibrils (probably due to electrostatic interactions) [63], and can be used to analyze of their degradation [64]), increased, which may indicate an increase in the number/size of protein aggregates in the sample despite the degradation of amyloid fibrils (Figure 2H,P). In can be noted that the sample contains alpha-B-crystallin in both bound to amyloids and free states. The results obtained in the literature studying the interaction of alpha-B-crystallin with fibrils formed from Abeta-peptide (1–42) in a 1/1 ratio indicate that approximately 50% of the chaperone is in a bound to fibrils state [30]. In this regard, we analyzed the binding of ANS to free alpha-B-crystallin (Figure S5). It turned out that the fluorescence intensity of the dye in the presence of a free chaperone was significantly lower than the fluorescence intensity of the dye bound to amyloid fibrils (by an order of magnitude). This means that the change in the ANS fluorescence intensity that we observed during the experiment mainly reflected changes in the structure of amyloid fibrils induced by alpha-B-crystallin (as they were caused precisely by changes in the properties of the bound to fibrils dye fraction).

Approximately 5 days after the start of the experiment, the process of degradation of the fibrils formed from lysozyme and beta-2-microglobulin was completed, which was confirmed by the stabilization of the intrinsic characteristics of the samples (Figure 2). As evidenced by TEM, both samples contained fibrils and disordered aggregates that did not degrade completely. Additionally to the TEM data (Figure 1), the presence of amyloid fibrils in the samples was confirmed by the relatively high fluorescence of ThT after the end of the degradation process (Figure 2G,O), as well as by the relatively high content of beta-sheet structures (Table 1).

Despite the similar mechanisms of alpha-B-crystallin-induced degradation of the studied amyloid fibrils, some differences were found in this process. In particular, it turned out that the rate of beta-2-microglobulin fibrils’ decompaction was higher than that of lysozyme fibrils. According to the TEM data, 20 min after the beginning of the experiment, a significant part of the beta-2-microglobulin fibrils was decompacted (Figure 1, bottom row). In contrast, the lysozyme fibrils were decompacted to a much lesser extent during this time (Figure 1, top row). The sample predominantly consisted of fibrils clots bordered by alpha-B-crystallin complexes with a small admixture of degraded non-fibrillar aggregates. After 4 h from the beginning of the experiment, a noticeable amount of large fibrillar clots was still detected in the sample with lysozyme aggregates, but the number of disordered aggregates and the degree of their decompaction increased. After five days from the beginning of the experiment, the number of non-fibrillar aggregates noticeably prevailed over the number of fibrillar structures.

It should also be noted that, after the end of degradation, in addition to disordered aggregates, fibrillar structures were also observed in both samples; moreover, in the sample with lysozyme aggregates, there were noticeably more fibrillar structures than in the sample with beta-2-microglobulin aggregates. The most obvious reason for the different efficiency and rate of alpha-B-crystallin action on the studied amyloid fibrils is the differences in the amino acid sequence and stability of the amyloidogenic proteins. Alternatively, the observed differences may arise from the different degree of aggregation and thickness of amyloid fibrils, that is, the different accessibility of chaperone binding sites on the fibrils. In order to test these hypotheses, we investigated the effect of alpha-B-crystallin on lysozyme amyloid fibrils, which have a significantly lower tendency to clustering than the fibrils already studied by us (prepared in buffer with neutral pH in the presence of GdnHCl.

### 2.4. The Rate of Amyloid Fibrils Degradation Induced by Alpha-B-Crystallin Depends to A Greater Extent on Their Thickness and Ability to Cluster Than on the Amino Acid Sequence of the Amyloidogenic Protein

**Figure 3:**
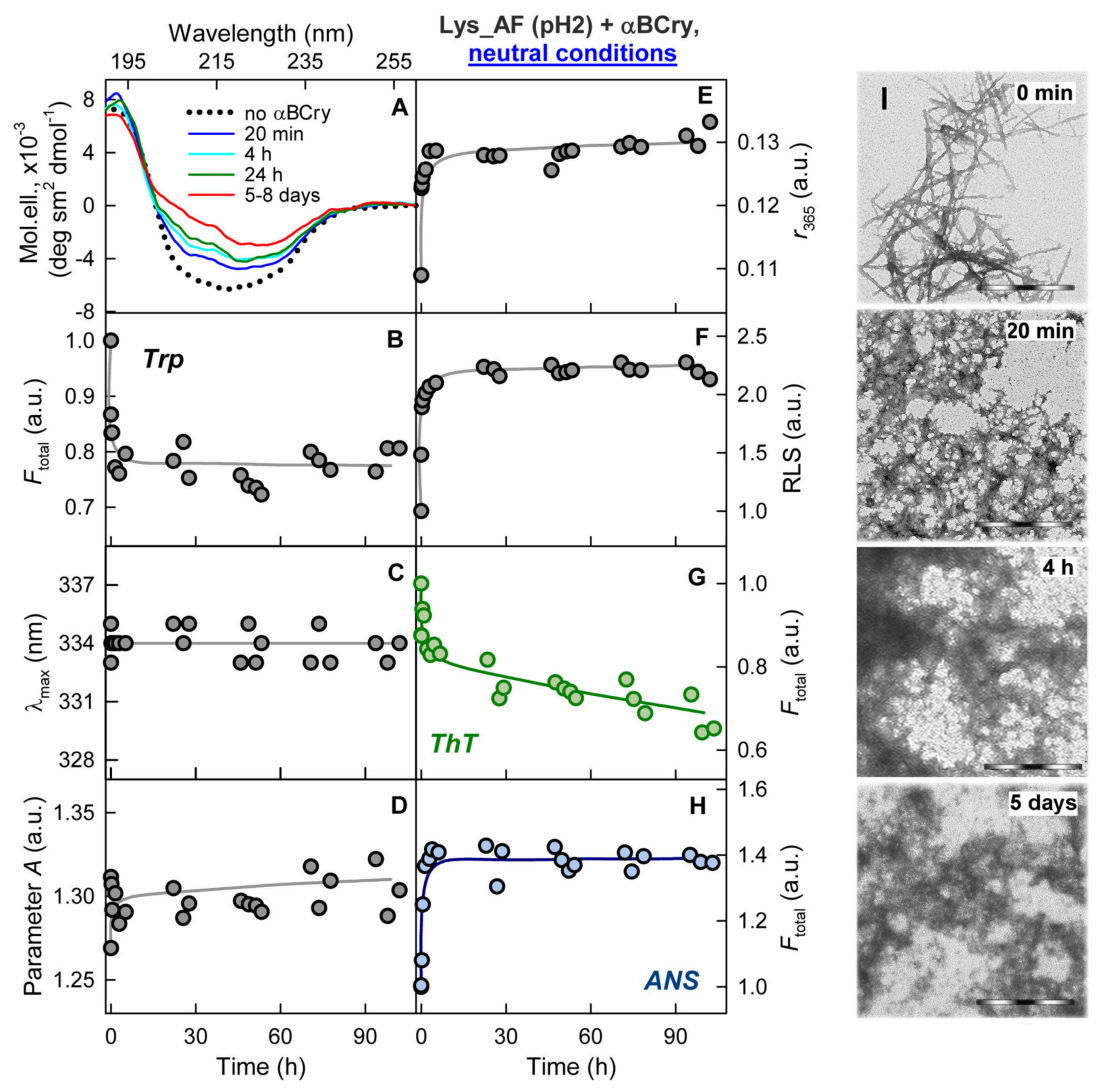
Alpha-B-crystallin-induced degradation of lysozyme amyloid fibrils without a high tendency to clustering under neutral conditions. Amyloid fibrils were prepared from lysozyme at pH 2 (Lys_AF (pH 2)). Figure shows alpha-B-crystallin induced changes in (**A**) far UV CD spectra of amyloid fibrils; (**B**) integrated fluorescence intensity of amyloid fibrils at excitation wavelength of 295 nm; (**C**) wavelength of the fluorescence spectrum maximum of amyloid fibrils; (**D**) parameter *A* of amyloid fibrils, λ_ex_ = 295 nm; (**E**) fluorescence anisotropy (*r*) of amyloid fibrils, λ_ex_ = 295 nm, λ_em_ = 365 nm; (**F**) Rayleigh Light Scattering (RLS) of amyloid fibrils; (**G**) integrated fluorescence intensity of ThT in the presence of amyloid fibrils; (**H**) integrated fluorescence intensity of ANS in the presence of amyloid fibrils. The values at the panels (**B**,**F**,**G**,**H**) are normalized to the values for fibrils in the absence of alpha-B-crystallin. (**I**) TEM images of degradation of amyloid fibrils in the presence of alpha-B-crystallin. The time after the start of chaperone exposure to fibrils is indicated to the right of the panels. The experiments were conducted under physiological conditions (pH 7.4) and the molar ratio of fibrils to alpha-B-crystallin was kept at 1:0.5. Scale bars are equal to 1 µm. The standard error of the mean is determined for a confidence interval of 0.95 and does not exceed 10%.

Based on the literature data, the acidic conditions of fibrillogenesis were successfully applied for preparation of lysozyme amyloid fibrils with a relatively low tendency to clustering (see Materials and methods) [43]. The prepared fibrils were transferred into a buffer with pH 7.4, and their stability was monitored during the experiment. It was noted that the secondary structure of these amyloid fibrils was markedly different from the secondary structure of the already investigated lysozyme amyloid fibrils (Figure 3A, Table 1). The RLS values of the lysozyme fibrils prepared at pH 2 were lower than those of the lysozyme fibrils obtained at pH 7, and even lower than the characteristics of the beta-2-microglobulin fibrils (Table 2). This fact, as well as TEM data, confirmed the smaller size of fibrillar clusters compared to lysozyme fibrils obtained under other conditions (in buffer with neutral pH in the presence of GdnHCl) (Figure 3I).

The mechanism of newly prepared amyloid fibril degradation was, as expected, similar to the mechanism that we discovered earlier (Figure 3). In the presence of alpha-B-crystallin, lysozyme amyloid fibrils were decompacted and fragmented, and the structure of the amyloid-forming proteins became less ordered (Table 1). It turned out that these amyloid fibrils degraded as fast as beta-2-microglobulin amyloid fibrils. Within 20 min after the start of the experiment, the sample contained predominantly unstructured aggregates (Figure 3I), and 5 days after the beginning of the experiment, the sample represented an equilibrium system of unstructured aggregates of various sizes (resulting from their fragmentation).

The results of the experiment allowed us to conclude that the mechanism of degradation of amyloid fibrils with different structures and properties, induced by alpha-B-crystallin under physiological conditions, was identical. It was shown that the efficiency and rate of the action of this chaperone-like protein on amyloid fibrils was mainly determined not by the amino acid sequence of the amyloid-forming protein, but by the morphology and degree of clustering of amyloid fibrils, that is, by the availability of binding sites for alpha-B-crystallin.

### 2.5. The Effect of Alpha-B-Crystallin on Amyloid Fibrils Does Not Change Their Resistance to Degradation

**Figure 4:**
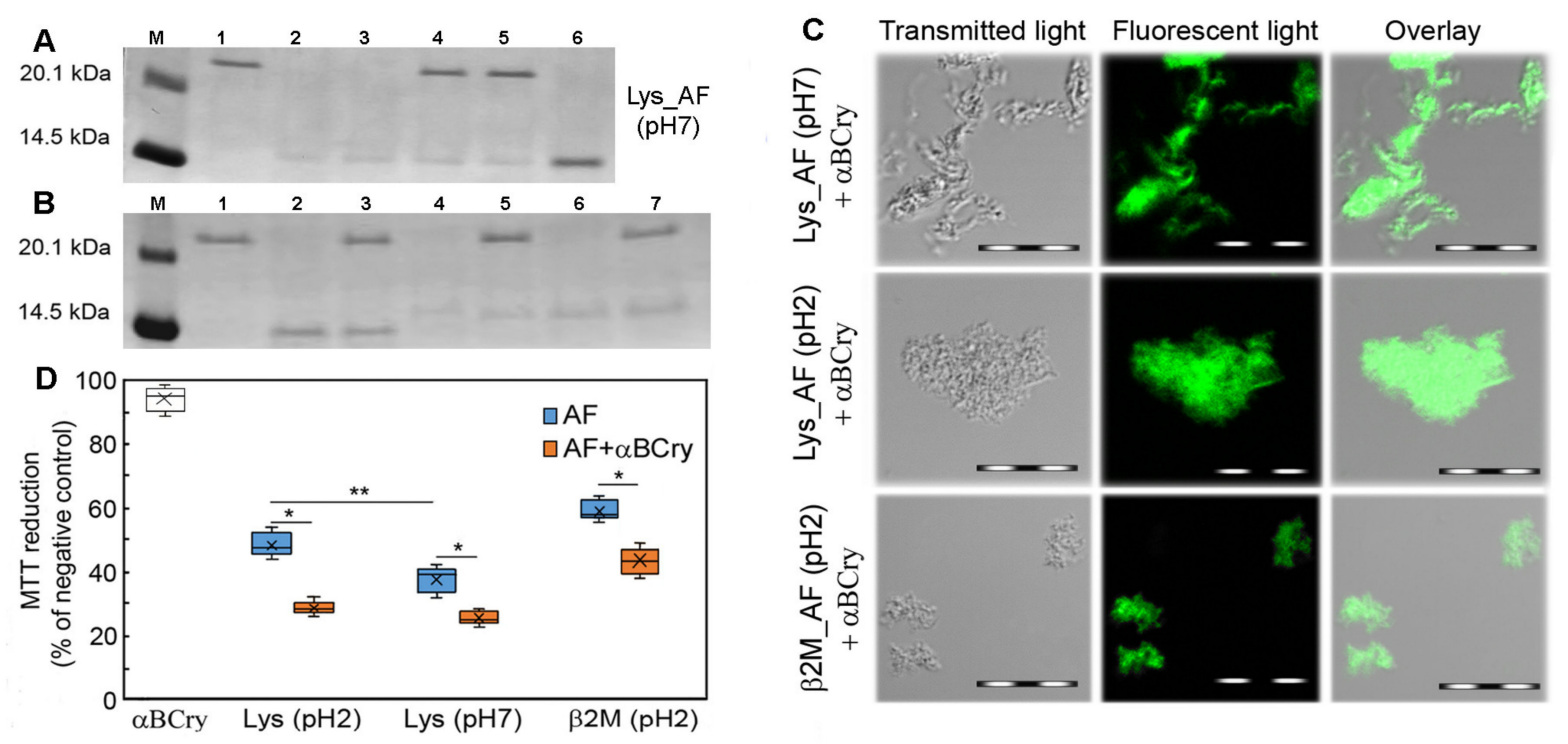
Stability and cytotoxicity of amyloid fibrils after the effect of alpha-B-crystallin under neutral conditions. The conditions of fibrils preparation are indicated in brackets. (**A**) Detergent resistance of the lysozyme amyloid fibrils prepared at pH 7 ((Lys_AF (pH 7)) before and after the effect of alpha-B-crystallin under physiological conditions detected by gel electrophoresis. Samples were loaded into the wells of the following lanes: (M) marker proteins, (1) alpha-B-crystallin, (2, 3) mature amyloid fibrils in cold and hot 2% SDS, respectively, (4, 5) mature amyloid fibrils after the influence of alpha-B-crystallin in cold and hot 2% SDS, respectively, (6) monomeric lysozyme. (**B**) Resistance of the amyloid fibrils to hot 2% SDS before (2, 4, 6) and after (3, 5, 7) the effect of alpha-B-crystallin detected by gel electrophoresis. Samples were loaded into the wells of the following lanes: (M) marker proteins; (1) alpha-B-crystallin; (2 and 3) beta-2-microglobulin amyloid fibrils (prepared at pH 2) before and after the effect of alpha-B-crystallin, respectively; (4 and 5) lysozyme amyloid fibrils (prepared at pH 7) before and after the effect of alpha-B-crystallin, respectively; (6 and 7) and lysozyme amyloid fibrils (prepared at pH 2), before and after the effect of alpha-B-crystallin, respectively. (**C**) Visualization by confocal microscopy of amyloid fibrils formed from different proteins exposed to alpha-B-crystallin. Transmitted light images (left column), confocal fluorescence images (middle column), overlay of the images (right column) are presented. Scale bars are 10 µm. (**D**) MTT assay results of HeLa cells treated with aggregates resulting after degradation of amyloid fibrils formed from beta-2-microglobulin (β2M) and lysozyme (Lys (pH 7) and Lys (pH 2), respectively) by alpha-B-crystallin (αBCry) under physiological conditions. Values are given as percentage of MTT signal compared with controls. * *p* = 0.01, ** *p* < 0.05 (*n* = 5, ANOVA, Tukey’s post hoc test).

Since the action of alpha-B-crystallin led to a change in the structure of amyloid fibrils, we were interested in whether the stability of mature amyloid fibrils changed after chaperone exposure. The resistance of these aggregates to treatment with sodium dodecyl sulfate (SDS) ionic detergent and boiling at 100 °C was analyzed. Figure 4A shows that lysozyme amyloid fibrils obtained at neutral pH are equally stable to detergents both at room temperature and after boiling (Figure 4A, lanes 2 and 3, respectively). Only a weak band was observed corresponding to the molecular weight of monomeric lysozyme (the rest of the protein loaded onto the lane, apparently, remained in an aggregated form and did not enter the gel). As a control, a strong band corresponding to the monomeric lysozyme at the same concentration as the amyloid fibrils is shown (lane 6). It turned out that lysozyme fibrils obtained at a neutral pH were the most stable to detergents and boiling among the three types of studied fibrils. The band corresponding to the monomeric protein for the sample with these fibrils has the lowest intensity (Figure 4B, lane 4) compared to fibrils obtained from lysozyme at acidic pH (Figure 4B, lane 6) and from beta-2-microglobulin (Figure 4B, lane 2). This is in line with a lesser influence of the alpha-B-crystallin on these fibrils. Presumably, the high stability of lysozyme fibrils obtained at a neutral pH of the solution is due to their high clusterization.

Surprisingly, the results of our experiment demonstrated that, for all amyloid fibrils after chaperone action, bands of monomeric proteins remained the same as without any exposure (Figure 4A, lanes 2 and 4, 3 and 5; Figure 4B, lanes 2 and 3, 4 and 5, 6 and 7). These data emphasize that the disordered aggregates formed from amyloid fibrils in the presence of alpha-B-crystallin under neutral conditions are almost as stable as corresponding amyloid fibrils. Interestingly, according to our results, these aggregates also retained a significant proportion of the beta-sheet structure and were intensely stained with ThT (Figure 4C). Thus, in the presence of alpha-B-crystallin under physiological conditions, amyloid fibrils are transformed to aggregates that retain some properties of amyloid fibrils. In this regard, the intriguing question is whether the toxicity of amyloid fibrils decreases after alpha-B-crystallin exposure.

### 2.6. The Alpha-B-Crystallin Action on Amyloid Fibrils Can Reduce Cell Viability in the In Vitro System

To assess the effect on the cells of protein aggregates treated with the chaperone, we determined the metabolic activity of HeLa cells by the MTT colorimetric assay. This test makes it possible to evaluate the metabolic activity of cells by the amount of formazan recovered from 3-(4,5-Dimethylthiazol-2-yl)-2,5-diphenyltetrazolium bromide by cellular oxidoreductase enzymes [65]. We showed that the presence of native alpha-B-crystallin did not lead to changes in cell viability (Figure 4D). At the same time, all tested amyloid fibrils showed reliable cytotoxicity. Unexpectedly, in the presence of amyloid fibrils degraded by alpha-B-crystallin under physiological conditions, an additional decrease in the MTT signal intensity was observed (*p* = 0.01). Interestingly, despite the fact that in our experiments mature lysozyme fibrils with a lower tendency to clustering (obtained at pH 2) had a lesser effect on the vital activity of cells than fibrils prepared at pH 7, after their treatment with alpha-B-crystallin, this effect was leveled. Thus, it was shown that as a result of the alpha-B-crystallin exposure, there was an increase (in some cases more than 40%) in the cytotoxicity of mature amyloid fibrils.

In order to suggest the reason for the obtained effects, it is necessary to pay attention to the fact that the toxicity of mature amyloid fibrils is largely determined by changes in cell membranes: their excessive stabilization or destabilization when interacting with amyloid aggregates [66]. It can be assumed that, under the action of alpha-B-crystallin, the system is enriched with a more toxic component due to the decompaction of amyloid fibrils and a partial decrease in the ordering of their secondary structure, as a result of which their affinity for cell membranes increases. It is important to note that the discovered effects are shown for amyloid fibrils with different morphologies and prepared under different conditions, which confirms the universality of this effect. It is obvious that such activity of chaperones in vivo can contribute to a decrease in the functional activity of cells.

### 2.7. External Influences Can Lead to a Change in The Mechanism of Amyloid Fibrils Degradation Induced by Alpha-B-Crystallin

Decompaction of amyloid fibrils induced by alpha-B-crystallin was observed by us in physiological conditions optimal for the chaperone to work. However, according to the literature, pathological accumulation of amyloid fibrils in the body can occur as a result of stress. In vitro, these effects are simulated using various external factors, such as an increase in temperature, the presence of denaturing agents, a decrease in the pH of a solution, etc. It is shown in [30] that although the optimal pH for the functioning of alpha-B-crystallin lies in the range from 7 to 8, even at acidic pH this chaperone inhibits the growth of amyloid fibrils formed from Abeta-peptide and beta-2-microglobulin. In this regard, to simulate stress conditions that do not inhibit the activity of the chaperone and, at the same time, induce the formation of amyloid fibrils, we decided to increase the acidity of the solution to pH 2 [44,67]. It should be noted that a low pH is characteristic of organs where amyloid fibrils studied by us are accumulated (kidney, stomach, intestines, etc.) [68,69,70,71].

**Figure 5:**
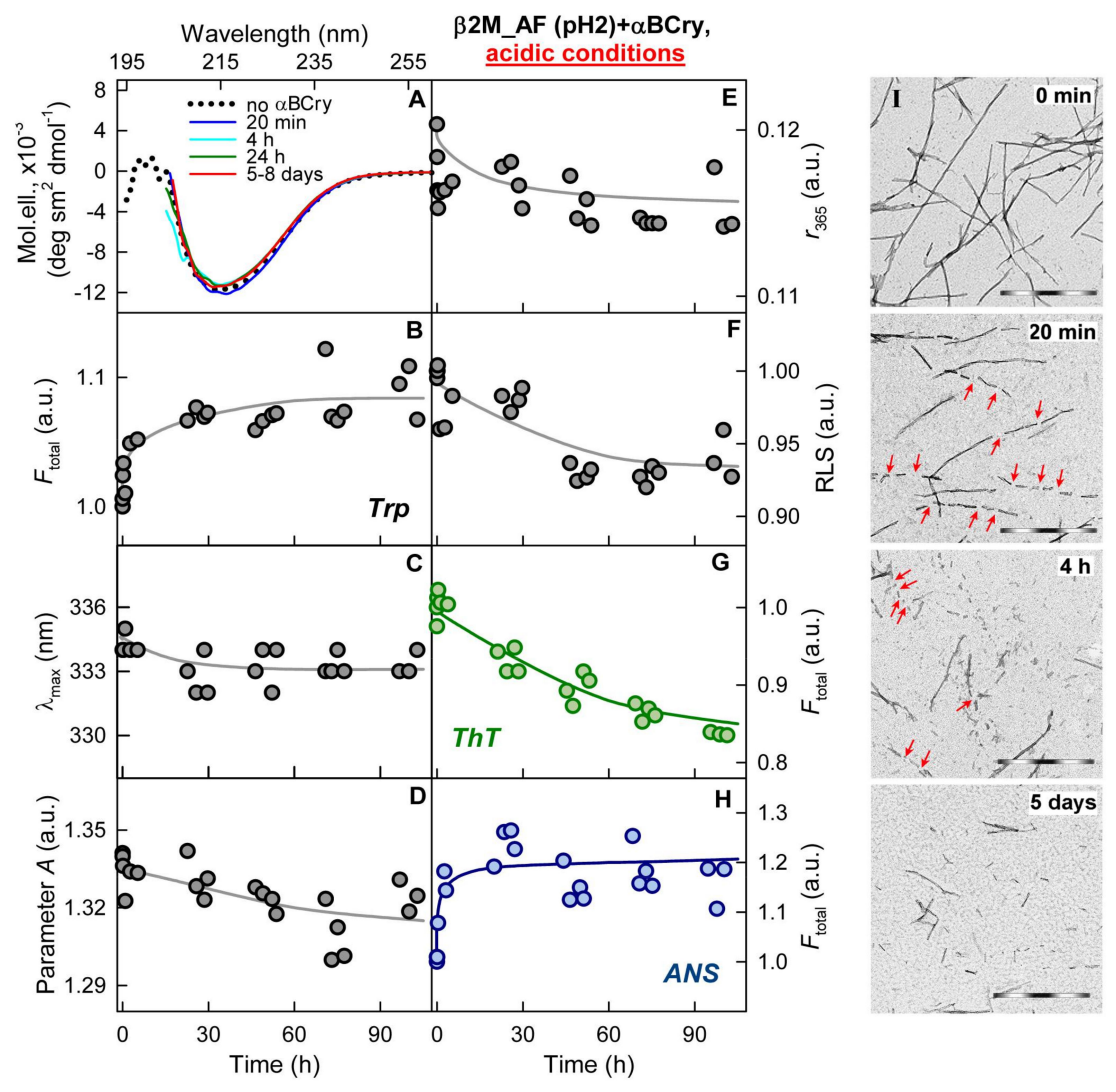
Alpha-B-crystallin-induced fragmentation of beta-2-microglobulin amyloid fibrils under acidic conditions. Amyloid fibrils were prepared from beta-2-microglobulin at pH2 (β2M_AF (pH2)). Figure shows alpha-B-crystallin induced changes in (**A**) far UV CD spectra of amyloid fibrils; (**B**) integrated fluorescence intensity of amyloid fibrils at excitation wavelength of 295 nm; (**C**) wavelength of the fluorescence spectrum maximum of amyloid fibrils; (**D**) parameter *A* of amyloid fibrils, λex = 295 nm; (**E**) fluorescence anisotropy (*r*) of amyloid fibrils, λex = 295 nm, λem = 365 nm; (**F**) Rayleigh Light Scattering (RLS) of amyloid fibrils; (**G**) integrated fluorescence intensity of ThT in the presence of amyloid fibrils; (**H**) integrated fluorescence intensity of ANS in the presence of amyloid fibrils. The values at the panels (**B**,**F**,**G**,**H**) are normalized to the values for fibrils in the absence of alpha-B-crystallin. (**I**) TEM images of degradation of amyloid fibrils in the presence of alpha-B-crystallin. The time after the start of chaperone exposure to fibrils is indicated to the right of the panels. Red arrows show the cutting sites of the fibril in the presence of the chaperone. The experiments were conducted under acidic conditions (pH 2.0) and the molar ratio of fibrils to alpha-B-crystallin was kept at 1:0.5. Scale bars are equal to 1 µm. The standard error of the mean is determined for a confidence interval of 0.95 and does not exceed 10%.

First of all, we checked that at acidic pH the size of alpha-B-crystallin oligomers, their morphology and secondary structure without amyloid fibrils did not change, at least at the time during which the experiment with amyloid fibrils was carried out (Figure S3). To obtain amyloid fibrils of beta-2-microglobulin at acidic pH, the protocol already used in this work was applied without the subsequent transfer of fibrils to a buffer solution with a neutral pH. As expected, these fibrils were highly stable under the conditions of their preparation and were long thin fibers without a high tendency to clustering (Figure 5I).

We showed that, as in the case of neutral conditions, alpha-B-crystallin aggregates interacted with the surface of amyloid fibrils along their long axis at acidic pH (Figure S4). A treatment of beta-2-microglobulin amyloid fibrils for 20 min with alpha-B-crystallin did not result in significant changes in the fibrillar morphology of amyloids; however, we found that some fibrils were fragmented (Figure 5I). After 4 h and up to 5 days after the beginning of the experiment, we observed further fragmentation of amyloid fibrils into shorter aggregates with preservation of their fibrous structure. During this time, we also observed a slight change in the intrinsic photophysical characteristics of the proteins that form fibrils (Figure 5B–E), a decrease in light scattering (Figure 5F) and the intensity of ThT fluorescence (Figure 5G). This confirmed the character of the degrading effect of alpha-B-crystallin in the acidic conditions, detected by TEM.

It was unexpected that the increase in the acidity of the solution led to a change in the mechanism of alpha-B-crystallin action on fibrils. We did not observe “fluffing” and decompaction of amyloids with a change in the ordering of their structure, but only their degradation into shorter fragments was revealed. This was evidenced by the opposite changes in the RLS of fibrils treated by the chaperone at acidic and neutral pH (Figure 2N and Figure 5F) and the differences in the change in the CD spectra (Figure 2I and Figure 5A). While at neutral pH, alpha-B-crystallin induced an increase in the RLS of fibrils and lowering of the ordering of the aggregate structure, at acidic pH, the RLS of fibrils decreased, and the secondary structure of aggregates remained practically unchanged.

**Figure 6:**
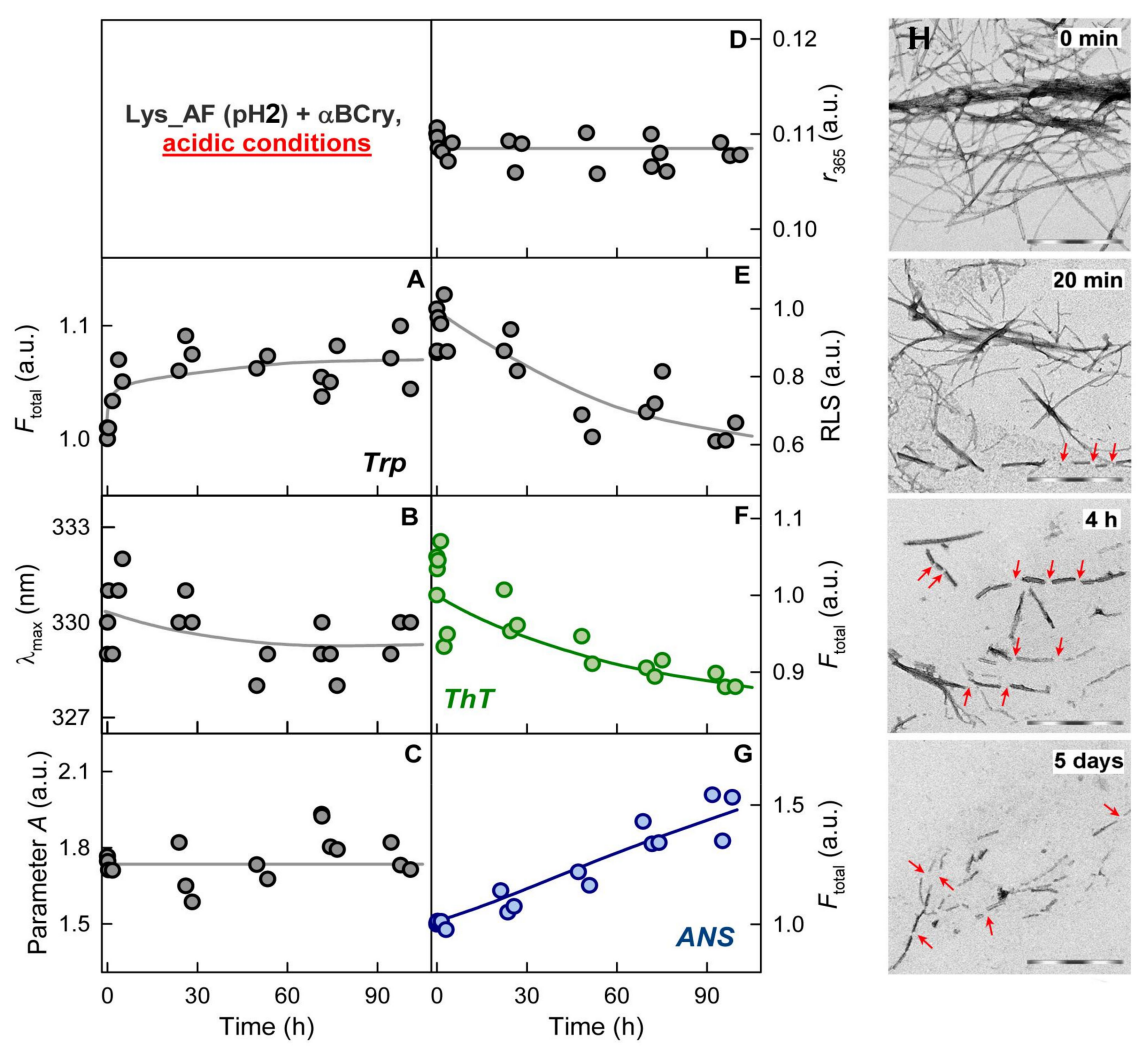
Alpha-B-crystallin-induced fragmentation of lysozyme amyloid fibrils under acidic conditions. Amyloid fibrils were prepared from lysozyme at pH 2 (Lys_AF (pH2)). Figure shows alpha-B-crystallin induced changes in (**A**) integrated fluorescence intensity of amyloid fibrils at excitation wavelength of 295 nm; (**B**) wavelength of the fluorescence spectrum maximum of amyloid fibrils; (**C**) parameter *A* of amyloid fibrils, λex = 295 nm; (**D**) fluorescence anisotropy (*r*) of amyloid fibrils, λ*_ex_* = 295 nm, λ*_em_* = 365 nm; (**E**) Normalized Rayleigh Light Scattering (RLS) of amyloid fibrils; (**F**) integrated fluorescence intensity of ThT in the presence of amyloid fibrils; (**G**) integrated fluorescence intensity of ANS in the presence of amyloid fibrils. The values at the panels (**A,E,F,G**) are normalized to the values for fibrils in the absence of alpha-B-crystallin. (**H**) TEM images of degradation of amyloid fibrils in the presence of alpha-B-crystallin. The time after the start of chaperone exposure to fibrils is indicated to the right of the panels. Red arrows show the cutting sites of the fibril in the presence of the chaperone. The experiments were conducted under acidic conditions (pH 2.0) and the molar ratio of fibrils to alpha-B-crystallin was kept at 1:0.5. Scale bars are equal to 1 µm. The standard error of the mean is determined for a confidence interval of 0.95 and does not exceed 10%.

To find out whether the effect of alpha-B-crystallin on fibrils at acidic pH depends on the amino acid sequence of an amyloidogenic protein, a similar experiment in the same conditions was carried out with amyloid fibrils prepared from lysozyme by the special protocol [43]. As we expected, the effect of alpha-B-crystallin on lysozyme amyloid fibrils at acidic pH was similar to its effect on beta-2-microglobulin amyloid fibrils under the same conditions. We observed small changes in the intrinsic characteristics of fibril-forming proteins (Figure 6A–D), and a decrease in RLS (Figure 6E) and ThT fluorescence intensity (Figure 6F), which confirmed the degradation of amyloid fibrils. A decrease in RLS (Figure 6E) and TEM data (Figure 6H) showed that, at acidic pH, alpha-B-crystallin cut lysozyme amyloid fibrils without altering their fibrous structure, as in the case of beta-2-microglobulin fibrils. Interestingly, for both types of fibrils, an increase in the intensity of ANS fluorescence was shown during the degradation of amyloid fibrils (Figure 5H, Figure 6G). Perhaps this is due to the formation of some oligomeric species in the sample, with which ANS is able to bind. According to the literature, the generation of short fibrils and oligomeric species increases amyloid seeding capacity, as well as enhancing the ability of fibril samples to disrupt membranes and to reduce cell viability in vivo [31,72,73].

The obtained results allowed us to conclude that a change in the normal conditions of alpha-B-crystallin functioning leads to a change in the mechanism of its effect on amyloids formed from various amyloidogenic proteins. Depending on the experimental conditions, the chaperone can promote both the transformation of amyloid fibrils into swollen and less ordered aggregates, as well as the fragmentation of amyloid fibers with the preservation of their ordered secondary structure enriched by beta-sheets. Unexpectedly, the degradation of mature amyloid fibrils induced by alpha-B-crystallin, which belongs to the cellular machinery preventing protein aggregation, can lead to an aggravation of amyloidosis both in normal conditions and under the influence of external factors.

## 3. Discussion

**Scheme 1:**
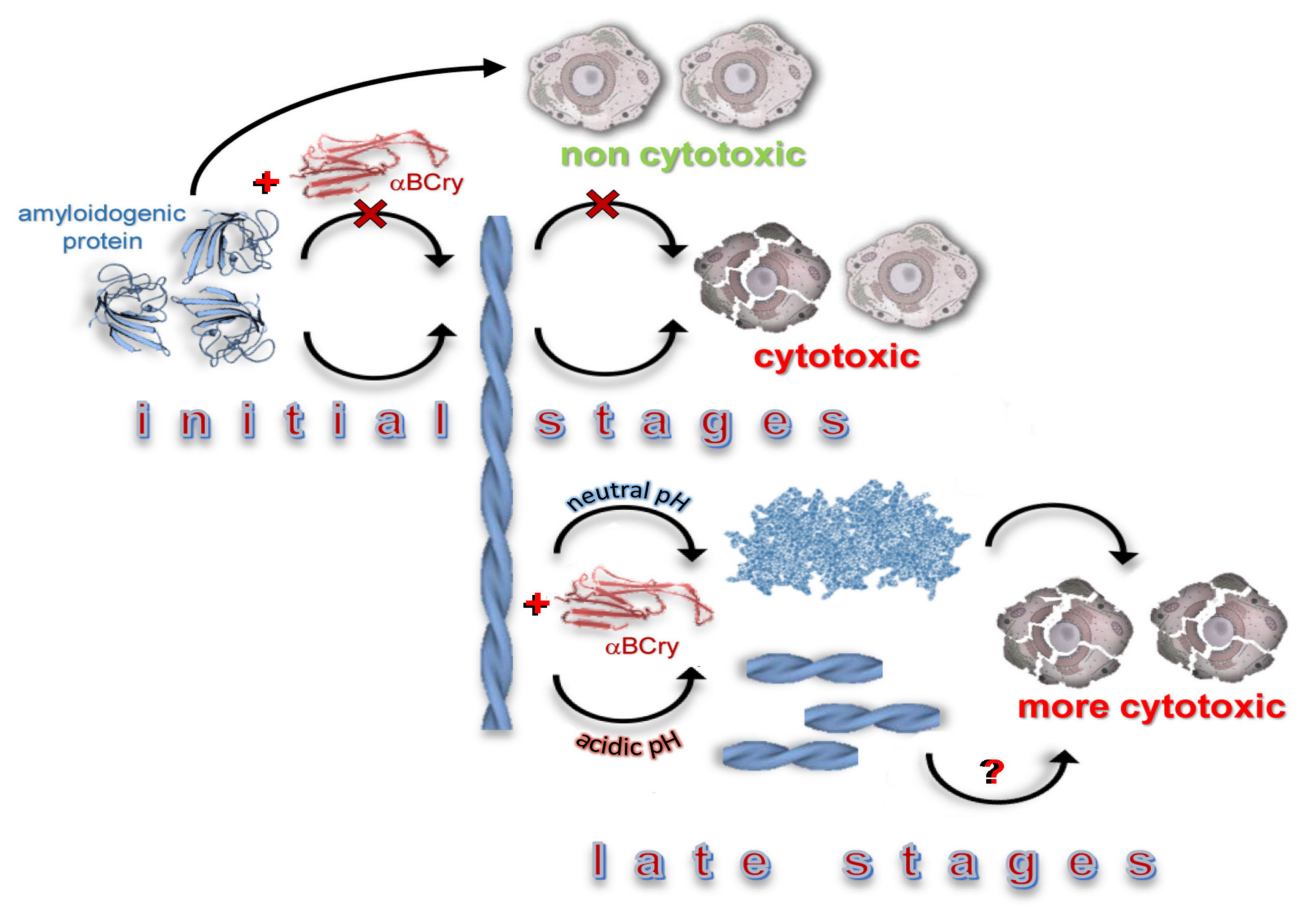
Role of alpha-B-crystallin in amyloidosis. Scheme shows the ability of alpha-B-crystallin to inhibit the formation of pathological amyloid fibrils [20,36,37]. This ability allowed early proposal of the chaperones-based therapy of amyloidosis on their initial stages (see, for example, the review [24]). However, since these diseases are often diagnosed at the later stages when a large amount of amyloids is already accumulated in the patient’s body, we pay attention to chaperones’ influence on mature amyloid fibrils. Scheme shows the various mechanisms of chaperone-induced degradation of the mature amyloids. Under physiological conditions, alpha-B-crystallin induces “fluffing” and decompactization of amyloid fibrils, which leads to a partial decrease in their structural ordering; however, it does not reduce their stability and can noticeably increase their cytotoxicity. Some external effects (for example, increase of medium acidity) could lead to a change in the mechanism of chaperone-induced fibrils’ degradation: amyloid fibers are fragmented without changing their secondary structure and properties. It was proposed in the literature that such an effect can lead to the generation of “seeds” for new bona fide amyloid fibrils, and can accelerate the accumulation of amyloids in the body, as well as enhance the ability of fibrils to disrupt membranes and to reduce cell viability [72,73]. This problem requires detailed further investigation.

The results of our work indicate that the protein with the chaperone activity alpha-B-crystallin not only inhibits the growth of amyloid fibrils, but also induces the degradation of mature amyloids, reducing the ordering of these protein aggregates under physiological conditions (Scheme 1). This fact, at first glance, seems to be an additional positive effect of alpha-B-crystallin in terms of amyloidosis treatment. However, we found that the action of alpha-B-crystallin does not change the stability of amyloid fibrils and increases their cytotoxicity (in some cases, significantly) (Scheme 1). Such activity of the chaperone in vivo will reduce the functional activity of cells and lead to an aggravation of amyloidosis.

At the same time, it was shown that a change in external conditions (an increase in the acidity of the environment), which often triggers the formation of amyloid fibrils, can lead to a change in the mechanism of alpha-B-crystallin action on amyloids: their fibers are fragmented without changing their secondary structure and properties. A similar mechanism of fibrils degradation induced by other external factors was previously described [72,73,74,75]. These works showed the negative consequences of amyloid fibrils fragmentation: a striking relationship was demonstrated between reduced fibril length caused by fibril fragmentation and enhanced ability of fibril samples to disrupt membranes and to reduce cell viability [72,73]. The fragmentation of amyloid fibrils by chaperones and the consequences of this process currently constitute a poorly understood problem.

The available data suggest that chaperones can act in the same way even under physiological conditions without any external influences. In particular, in the literature [31], it was shown that a specific combination of human Hsp70 disaggregase-associated chaperone components efficiently disassembled alpha-synuclein amyloid fibrils characteristic of Parkinson’s disease in vitro by their fragmentation. The authors of this work note that incomplete disassembly of amyloids by Hsc70/DNAJB1/Apg2 generates short fibrils and oligomeric species that are toxic and perhaps may even increase amyloid seeding capacity in vivo. The activity of chaperons and chaperon-like proteins therefore has Janus head features, the pathophysiological manifestation of which may depend on the balance of cellular proteostasis. Moreover, in the cited work [31], fibrils’ fragments were disassembled by the chaperone into monomers very quickly; however, in our work, we showed that fibril fragments of various lengths followed by alpha-B-crystallin exposure remained in the sample at least 5 days after chaperone addition. Thus, it can be assumed that the action of chaperones not only under physiological conditions, but also but also when these conditions change under the influence of external factors, can lead to the aggravation of amyloidosis.

Our results indicate that a therapeutic approach for slowing down and preventing amyloidosis, based on the increase in chaperones concentration by the inducing the synthesis of chaperones or using therapeutic drugs with chaperone activity, should be treated with caution. It should be borne in mind that for a specific therapeutic effect, it is necessary to select specific chaperones, and to study their effect not only on fibrillogenesis, but also on mature amyloid fibrils. It is highly likely that some chaperones will be effective in the early stages of the development of the disease, when the number of formed amyloid fibrils is not yet very significant, but will aggravate the situation by directly affecting the fibrils in the later stages of amyloidosis. In addition, when choosing one or another chaperone as a therapeutic agent, it is necessary to first assess the pathological consequences of stress effects that preceded the accumulation of amyloids in the body. Failure to comply with these requirements can lead not only to the absence of the chaperone therapy effect, but also to the progression of the disease.

## 4. Materials and Methods

### 4.1. Materials

The fluorescent dyes thioflavin T (ThT) “UltraPure Grade” (AnaSpec, Fremont, CA, USA) and 8-anilino-1-naphthalene sulfonate (ANS) from Serva (Heidelberg, Germany), isopropyl-beta-D-1-thiogalactopyranoside (IPTG; Fluka, Buchs, Switzerland), guanidine hydrochloride (GdnHCl), lysozyme and buffer components, 3-(4,5-dimethylthiazol-2-yl)-2,5-diphenyltetrazolium bromide (MTT) from Sigma (Saint Louis, MO, USA) were used without further purification. The Dulbecco’s Modified Eagle Medium (DMEM, glucose 4.5 g/L), fetal bovine serum (FBS), 0.25% Trypsin-EDTA were from Gibco (Thermo Fisher Scientific, Waltham, MA, USA). The culture flasks and 96-well plates (flat bottom) were from Corning (USA).

### 4.2. Alpha-B-Crystallin Isolation and Purification

The alpha-B-crystallin genes were amplified and cloned into a pET28a vector using NcoI and Xho I sites (the plasmid was commercially synthesized by Eurogen, Moscow, Russia). BL21(DE3) host cells (Invitrogen, Waltham, MA, USA) were transformed by pET28a plasmid for the expression of alpha-B-crystallin under IPTG inducible promoter. Bacterial cells were grown in Luria-Bertani (LB) broth supplemented with kanamycin. The expression of alpha-B-crystallin was initiated by 0.5 mM IPTG at 28 °C overnight. Alpha-B-crystallin isolation and purification was performed as described previously [76,77] with some modifications. The cell pellet was subjected to a freeze–thaw cycle. After sonication, DNA was degraded by the addition of DNase I to a final concentration of 2.5 mkg/L and the suspension was incubated for 45 min at 37 °C.

The crude cell lysate was fractionated by ammonium sulfate (the target protein precipitates by 40% ammonium sulfate). The subsequent steps of the alpha-B-crystallin purification included ion-exchange chromatography on a Mono Q 5/50 GL (GE Healthcare, Danderyd, Sweden) and size-exclusion chromatography on a Superose 12 10/300GL (GE Healthcare, Danderyd, Sweden). The protein was highly concentrated (up to 6–10 mg/mL) and stored in 25 mM TrisHCl, 0.1 mM EDTA, 50 mM NaCl, 0.1 mM PMSF, 1 mM DTT, pH 7.6. The purity of the protein was tested by SDS/PAGE in 12% polyacrylamide gels [78]. The protein concentration was calculated using an extinction coefficient at 280 nm of 14 100 M^−1^·cm^−1^ according to amino acids composition. Alpha-B-crystallin stability in different conditions and the retention of its characteristics for at least the time during which the experiment with amyloid fibrils was carried out were controlled (Figure S3).

### 4.3. Amyloid Fibrils Preparation

To prepare amyloid fibrils from beta-2-microglobulin, we followed the special method used in the work [42]. The recombinant beta-2-microglobulin was kindly provided by Mikhail M. Shavlovsky, Dmitry S. Polyakov and Rodion G. Sakhabeev (Department of Molecular Genetics, Institute of Experimental Medicine). Two different buffer and temperature conditions were used for obtaining the lysozyme amyloid fibrils: 100 mM KH_2_PO_4_-NaOH in the presence of 3 M GdnHCl (pH 7) at 57 °C and 20% acetic acid solution in the presence of 100 mM NaCl (pH 2) at 37 °C. Protein at a concentration of 2 mg/mL was incubated in a TS-100 Thermo-Shaker (Biosan, Warren, MI, USA) with constant agitation for 1 day (500 rpm). After the formation of mature amyloid fibrils for performing experiments in neutral physiological conditions, protein aggregates were firstly dialyzed against deionized water and then against 20 mM sodium phosphate buffer solution (pH 7.4). The fibril stability in different conditions and the retention of their characteristics for at least the time during which the experiment with alpha-B-crystallin was carried out were controlled (Figure S1).

### 4.4. Amyloid Fibrils Degradation by Alpha-B-Crystallin

Before starting the study of the effect of alpha-B-crystallin on amyloid fibrils, we analyzed the structure and photophysical properties of the chaperone under the conditions of the experiments. In particular, the photophysical properties of alpha-B-crystallin were studied in 20 mM sodium phosphate buffer solution (pH 7.4) and 100 mM Gly-HCl. (pH 2). 

The fibril concentration was 0.1 mg/mL in all experiments, and the measurements were performed in 20 mM sodium phosphate buffer solution (pH 7.4). The freshly thawed alpha-B-crystallin was added to the fibril solutions at a molar ratio of 0.5 (or 0.1) to 1. The measurements were started immediately after manual mixing of the fibril solutions with alpha-B-crystallin. According to the control experiments, the dead-time in these manual mixing-based experiments was about 4 s [46,79]. The characteristics and properties of the prepared samples were detected by a wide range of physicochemical approaches at room temperature for 8 days. To obtain TEM images, 10 μL of the sample was taken at different time intervals. It was shown that the recorded characteristics of the samples stopped changing 5 days after the start of the experiment.

To analyze the stability of mature fibrils and fibrils after the alpha-B-crystallin exposure, a sample buffer for denaturing electrophoresis containing sodium dodecyl sulfate (SDS) was added to 10 μL of each sample [78]. After boiling, the samples were loaded on 15% PAGE. Samples without boiling were prepared in a similar manner.

### 4.5. Transmission Electron Microscopy

Visualization of various aggregates and amyloid fibrils was carried out using a transmission electron microscope Libra 120 (Carl Zeiss, Jena, Germany). To prepare the samples for TEM, copper grids coated with formvar/carbon films (Electron Microscopy Sciences, Hatfield, PA, USA) were used. The grids with sample were stained by a 1% aqueous solution of uranyl acetate.

### 4.6. Confocal Microscopy

Visualization of the ThT-stained fibrillar structures was carried out using an Olympus FV 3000 confocal laser scanning microscope (Olympus, Tokyo, Japan) and oil immersion objective with a 60x magnification, numerical aperture NA 1.42 and laser with an excitation line of 405 nm.

### 4.7. Spectral Measurements

Samples for fluorescence studies were prepared as described in Section 4.4. The absorption spectra of the probes were collected using a U-3900H spectrophotometer (Hitachi, Tokyo, Japan). The light scattering was subtracted from the absorption spectra of amyloid fibrils and ThT in the presence of the fibrils using a standard procedure [80]. The concentrations of ThT and ANS and fibrils from lysozyme and beta-2-microglobulin were calculated based on molar extinction coefficients of *ε*_412_ = 31,600 M^−1^·cm^−1^, *ε*_350_ = 5000 M^−1^·cm^−1^, *ε*_280_ = 36,000 M^−1^·cm^−1^ and *ε*_276_ = 20,065 M^−1^·cm^−1^, respectively. In experiments with fluorescent probes, the value of absorbance of ThT and ANS was 0.5.

A Cary Eclipse spectrofluorimeter (Varian, Melbourne, Australia) was applied to collect fluorescence spectra. Fluorescence of the fibrils was excited at 295 nm. Parameter *A* = *I*_320_/I_365_, where *I*_32__0_ and *I*_365_ are the fluorescence intensities at the emission wavelengths of 320 and 365 nm, respectively, was measured for characterization of the position and form of the fluorescence spectra [71]. The value for parameter A was corrected, taking into account the instrument sensitivity. The anisotropy of tryptophan fluorescence was determined from:

(1) $$r=\frac{\left(I_{V}^{V}-GI_{H}^{V}\right)}{\left(I_{V}^{V}+2GI_{H}^{V}\right)}$$

where $I_{V}^{V}$ and $I_{H}^{V}$ are vertical and horizontal components of the fluorescence intensity excited by vertically polarized light, respectively, and $G=I_{V}^{H}/I_{H}^{H}$ is the coefficient that determines the different instrument sensitivity for the vertical and horizontal components of the fluorescence light, λ_em_ = 365 nm [81]. The Rayleigh light scattering (RLS) of samples with fibrils were measured with the same excitation and registration wavelength (295 nm). The RLS value of the chaperone molecules themselves was low enough, but it was used for the correction of RLS value of samples of amyloid fibrils in the presence of alpha-B-crystallin (Figure S5A).

Fluorescence of the dyes, ThT and ANS, was excited at a wavelength of 440 and 350 nm, respectively. The spectral slits width did not exceeded 5 nm in most of experiments. Increasing the slit widths did not influence the experimental results. The correction of fluorescence intensity on the primary inner filter effect was carried out using a previously elaborated approach [82]. When analyzing changes in the ThT and ANS intensities during the degradation of amyloid fibrils under the influence of alpha-B-crystallin, the fluorescence intensity of these dyes in the presence of chaperone without amyloids was taken into account (Figure S5B).

Samples for CD-spectroscopy were prepared as described in Section 4.4. To collect the CD spectra in the far UV-region of the samples we used a J-810 spectropolarimeter (Jasco, Tokyo, Japan) and a 0.1 cm cell. The measurement range was 200–260 nm. The recorded spectra were averaged over three scans. The CD spectrum of the samples was corrected, taking into account the signal of appropriate buffer. To assess the content of various elements of the secondary structure in amyloid-forming proteins, the CD spectra of fibrils in the presence of alpha-B-crystallin (measured at different time intervals after mixing the solutions) were corrected taking into account the CD spectrum of the chaperone. Alpha-B-crystallin remained stable throughout the experiment, as shown using EM and CD spectroscopy (see Figure S3). After the subtraction of the alpha-B-crystallin CD spectrum, the CD spectra of fibrils were analyzed by CDPro software according to Provencher’s method [45]. Three different regression methods (Selcon, Contin, and CDSSTR) and several basic sets of proteins with a known secondary structure (the sets include from 37 to 56 soluble, membrane, and denatured proteins with different content of the secondary structure) of the CDPro program package were applied for the secondary structure evaluation.

### 4.8. MTT Assay

HeLa cells were routinely cultured in DMEM-10% FBS supplemented with 50 μg/mL penicillin-streptomycin and 2 mM l-glutamine, and kept in a 5% CO_2_ humidified incubator at 37 °C. For the MTT assay, confluent HeLa cells were stripped from culture flasks with 0.25% Trypsin-EDTA, washed with DPBS and plated in 96-well coated culture plates at density of 3000 viable cells/well in 120 µl of culture medium. The cells were incubated for 24 h at 37 °C and 5% CO_2_ and amyloid aggregates were administered to cells. The final concentration of aggregates was 0.7 μM for lysozyme and 1 μM for b2M on a monomer basis. The toxicity was assessed by the MTT (Sigma-Aldrich, St. Louis, MO, USA) reduction inhibition assay based on the protocol described for the first time by Mosmann [83,84]. In all MTT experiments, the cells were treated with the aggregates for 24 h and then incubated for 3 h with 100 μL of DMEM without phenol red and FBS, containing 0.5 µg/µl MTT. A quantity of 100 μL of DMSO was added to each well and the samples were incubated at 37 °C to allow complete lysis. The absorbance values were determined at 595 nm with an automatic plate reader (Bio-Rad, Milan, Italy). The final absorption values were calculated by averaging 5 independent measurements of each sample and subtracting from this the average of the blank. Readings from the different conditions were expressed as percent viability with respect to controls containing equal amounts of alpha-B-crystallin in amyloid incubation buffer.

### 4.9. Statistical Analysis 

The spectral characteristics of fibrils were determined as a result of at least three independent experiments. The standard error of the mean is determined for a confidence interval of 0.95.

The results of the MMT assay are presented as median. All experiments were performed at least in triplicate. To test the sample data for normal distribution, the Kolmogorov–Smirnov test was used. Multiple group comparisons were processed using the one-way analysis of variance (ANOVA) method with Tukey’s post hoc test. The differences were considered significant at *p* < 0.05. Data were analysed using on-line calculator software (https://astatsa.com/OneWay_Anova_with_TukeyHSD/).

## Figures and Tables

**Table 1:** The changes of secondary structure content ^1^ of amyloid fibrils formed from different amyloidogenic proteins induced by alpha-B-crystalline under neutral conditions (pH 7.4).

Time of Fibrils Incubation in the Presence of αBCry	Lysozyme Fibrils (Prepared at pH 7)				Beta-2-Microglobulin Fibrils (Prepared at pH 2)				Lysozyme Fibrils (Prepared at pH 2)			
	α, %	β, %	T, %	U,%	α, %	β, %	T, %	U, %	α, %	β, %	T, %	U, %
0 min	2.6	48.3	18.1	31.0	7.3	43.9	26.1	22.7	14.8	32.0	23.1	30.1
20 min	2.9	39.6	18.8	38.7	6.2	38.4	26.3	29.1	11.9	32.4	21.5	34.2
4 h	2.7	38.4	18.1	40.8	6.7	38.5	22.5	32.3	8.0	32.9	22.2	36.9
1 day	2.7	38.3	18.0	41.0	5.5	38.6	21.0	34.9	7.3	31.0	22.1	39.6
5–8 days	2.6	38.0	18.6	40.8	4.5	38.5	21.3	35.7	4.9	32.0	21.8	41.3

Analysis was made on the basis of the far-UV circular dichroism (CD) spectra using the Provencher’s algorithm [45].

**Table 2:** Photophysical characteristics of amyloid fibrils formed from different amyloidogenic proteins and fluorescent probes bound to them.

Characteristic	Lysozyme Fibrils (Prepared at pH 7)	Beta-2-Microglobulin Fibrils (Prepared at pH 2)	Lysozyme Fibrils (Prepared at pH 2)
RLS	370 ± 7	204 ± 19	159 ± 15
*r*	0.16 ± 0.01	0.13 ± 0.01	0.11 ± 0.01
*A*	1.28 ± 0.04	1.47 ± 0.04	1.27 ± 0.04
*F*_total_·10^−3^	24.9 ± 0.8	10.4 ± 0.3	32.5 ± 1.0
*F*_total_ThT_·10^−3^	53 ± 1	21 ± 1	14 ± 1
*F*_total_ANS_·10^−3^	72.0 ± 2.8	8.7 ± 0.3	26.0 ± 0.8

RLS—Rayleigh Light Scattering; *r*—fluorescence anisotropy; parameter *A*, *F*_total_, *F*_total_ThT_ and *F*_total_ANS_ —integral fluorescence intensity of amyloid fibrils and bound to them ThT and ANS, respectively.

## Supplementary Materials

Supplementary materials can be found at https://www.mdpi.com/1422-0067/21/20/7659/s1. Figure S1–S5.
