# Supplementary material for: Alpha-B-Crystallin Effect on Mature Amyloid Fibrils: Different Degradation Mechanisms and Changes in Cytotoxicity
Source: Int J Mol Sci. 2020 Oct 16;21(20):7659. doi: 10.3390/ijms21207659 (PMC7589196; doi:10.3390/ijms21207659)
Supplement: Supplementary file 1 [file ijms-21-07659-s001.pdf]

Supplementary Materials

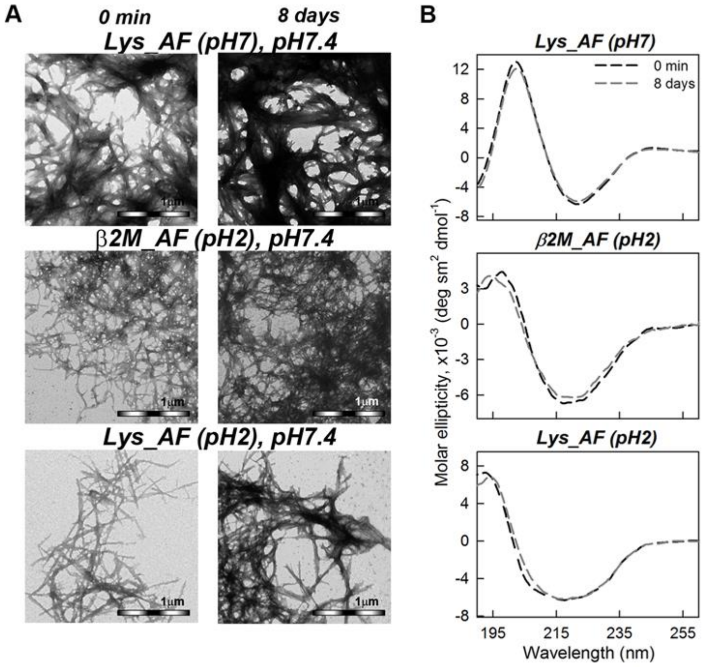

Figure S1. The stability of amyloid fibrils formed from lysozyme and beta-2-microglobulin in physiological conditions (pH 7.4) during 8 days (the time during which the experiment with alpha-B-crystallin was carried out). The morphology of amyloid fibrils was visualized by TEM (**A**). Scale bars are equal to 1 μm. Secondary structure of amyloid samples was controlled by CD spectroscopy (**B**).

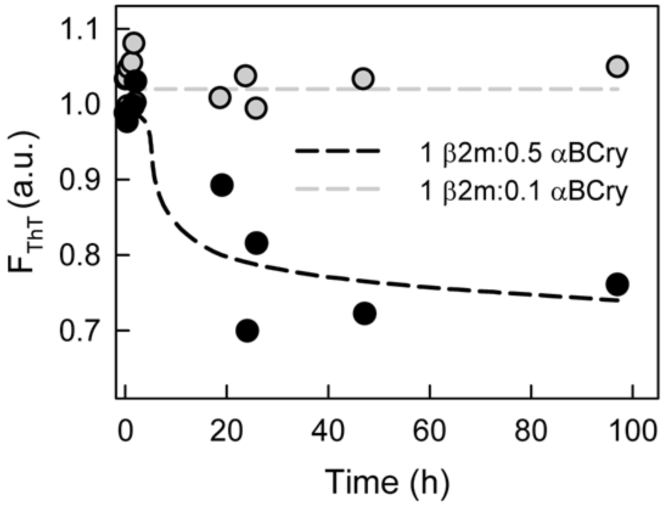

Figure S2. Selection of the molar ratio of alpha-B-crystallin and amyloid fibrils for experiment performing. The change in the integrated fluorescence intensity of ThT in the presence of amyloid fibrils formed from beta-2-microglobulin at pH 2 is shown. The experiments were conducted at physiological conditions (pH 7.4) and molar ratio of fibrils to alpha-B-crystallin was kept 1:0.1 and 1:0.5.

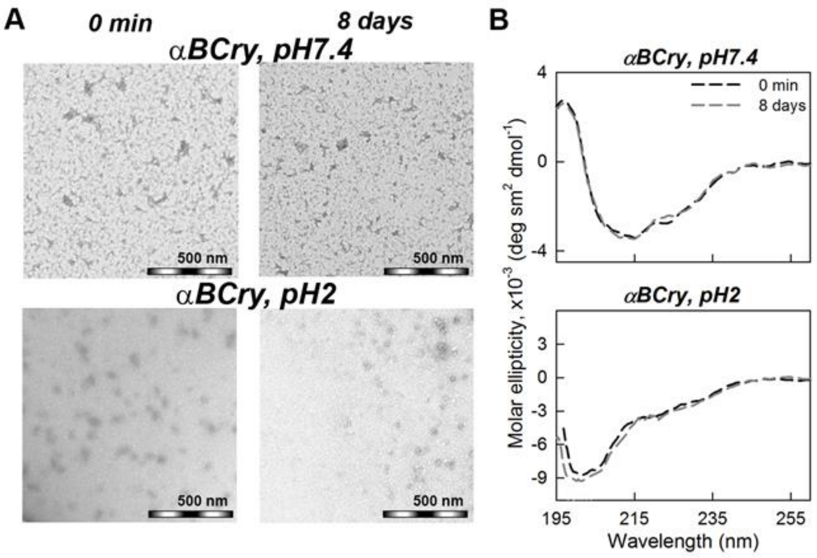

Figure S3. The stability of alpha-B-crystallin (αBCry) in physiological (pH7.4, top row) and acidic (pH2, bottom row) conditions during 8 days (the time during which the experiment with amyloid fibrils was carried out). The morphology of oligomers formed by αBCyr was visualized by TEM (**A**). Scale bars are equal to 500 nm. Secondary structure of the protein was controlled by CD spectroscopy (**B**).

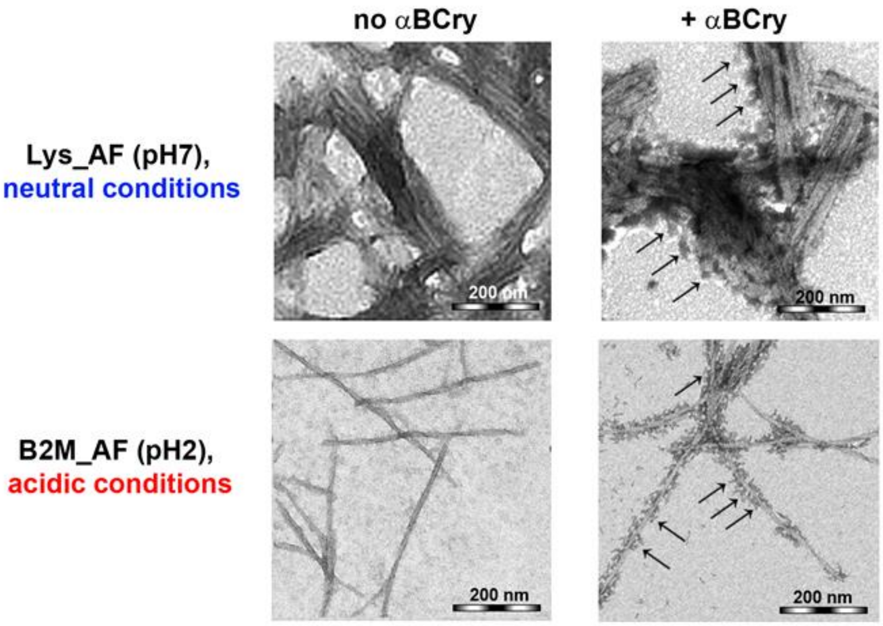

Figure S4. The binding of alpha-B-crystallin (αBCry) to amyloid fibrils in physiological (pH 7.4, top row) and acidic (Ph 2, bottom row) conditions. The TEM images of amyloid fibrils alone (left panels) and immediately after the addition of alpha-B-crystallin (αBCry) to the fibrils (right panels) illustrate that the chaperon aggregates interact with the surface of amyloid fibrils along their long axis (as shown by black arrows). Scale bars are equal to 200 nm.

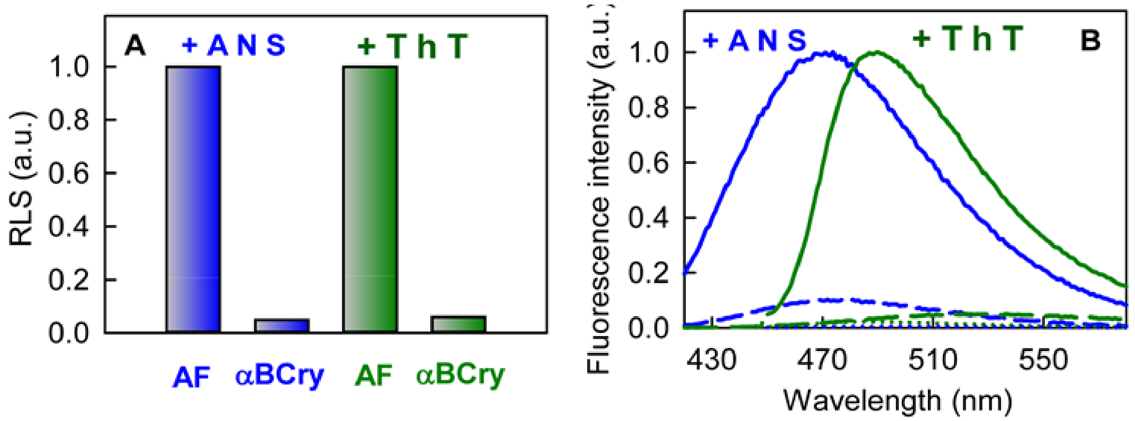

Figure S5. Comparison of alpha-B-crystallin and lysozyme amyloid fibrils photophysical properties and interaction with fluorescent probes under neutral conditions. (**A**) The Rayleigh Light Scattering (RLS) of amyloid fibrils (AF) and alpha-B-crystallin (αBCry) in the presence of ANS (blue color) and ThT (green color). (**B**) Fluorescence spectra of ANS (blue curves) and ThT (green lines) in free state (dotted lines) and in the presence of alpha-B-crystallin (dashed lines) and amyloid fibrils formed from lysozyme at pH 7 (solid lines). The standard error of the mean is determined for a confidence interval of 0.95 and does not exceed 10%.
